# Geomagnetic activity affects animal myocardial ischemia/reperfusion injury: an experimental-simulated study

**DOI:** 10.1007/s00484-024-02618-4

**Published:** 2024-01-10

**Authors:** Weiyu Chang, Xinli Chen, Yuan Yang, Yanglin Deng, Liang Dong, Hui Wu

**Affiliations:** 1 Clinical Pharmacy Center, First Affiliated Hospital of Kunming Medical University, Kunming, 650032 China; 2 Yunnan Observatories, Chinese Academy of Sciences, Kunming, 650216 China

**Keywords:** Inflammatory factors, Cardiovascular disease, Cardiac function

## Abstract

Numerous studies have shown that geomagnetic activity (GMA) contributes to the development and escalation of cardiovascular disease (CVD), as well as increased morbidity and mortality. However, the underlying molecular mechanisms and approaches for understanding GMA remain unclear. This study aimed to investigate the impact of GMA on oxidative stress and inflammatory responses. Myocardial ischemia/reperfusion injury (MI/RI) rat models were created under various geomagnetic field conditions. The range of cardiac function, markers of myocardial injury, inflammatory factors, and the TLR4/NF-κB signaling pathway were measured after the 24-h period. The findings showed that weak GMA significantly improved cardiac function in the MI/RI rat model and reduced the size of myocardial infarction and creatine kinase (CK) and lactic dehydrogenase (LDH) levels. Additionally, weak GMA enhanced superoxide dismutase (SOD) activity and decreased malondialdehyde (MDA) content. Furthermore, weak GMA significantly reduced the levels of the myocardial inflammatory cytokines interleukin-1 (IL-1), interleukin-6 (IL-6), and tumor necrosis factor-α (TNF-α). Conversely, the effects observed under severe GMA conditions were opposite to those observed under weak GMA. Western blot and qPCR analysis demonstrated that weak GMA led to a significant downregulation of TLR4, TRAF6, NF-κB, TNF-α, and MCP-1 in the MI/RI rat models. In contrast to weak GMA, severe GMA increased TLR4, TRAF6, NF-κB, and TNF-α expression. This study suggested that weak GMA had a limiting effect on MI/RI rat models, whereas severe GMA exacerbated injury in MI/RI rats. These effects were associated with oxidative stress and inflammatory responses and might potentially involve the TLR4/NF-κB signaling pathway.

## Introduction

The geomagnetic field is a barrier to life, playing a crucial role in the growth, development, and migration of various life forms. Human activities are intricately intertwined with the geomagnetic field (Gmitrov and Gmitrova 2004). Geomagnetic activity (GMA) is a disturbance of the geomagnetic field (GMF) caused by changes in electric currents in the magnetosphere and ionosphere. The main cause of such changes is the flow of perturbed solar wind that interacts with the geomagnetic field and adds energy to the magnetosphere–ionosphere current system (Akasofu and Chapman 1972). It is considered that the geomagnetic storms (GS), which are global changes in the geomagnetic field, have the greatest biological impact of all geomagnetic activities. Geomagnetic activity has been observed in human physiological systems spanning a variety of phenomena, including endocrine activity, brain activity, and heart activity (Okano 2008; Dimitrova et al. 2004; Khabarova and Dimitrova 2008). Many researchers have gathered clinical data from tens of thousands of cardiovascular patients worldwide, revealing that geomagnetic storms pose risks to heart health. There is a noticeable connection between overall mortality, mortality from cardiovascular disease (CVD), mortality from myocardial infarction (MI), and GMA (Vencloviene et al. 2013; Feigin et al. 2014; Kiznys et al 2020). Pathologically, research on the relationship between GMA and CVD mainly focuses on average heart rate and blood pressure indicators, which are often used in blood biology studies of the autonomic nervous system or cardiovascular function (Ozheredov et al. 2017). However, there have been relatively few studies on the mechanisms of GMA-induced cardiovascular changes.

The leading cause of many CVDs worldwide, ischemic heart disease (IHD), is now a significant contributor to death and morbidity (Barquera et al. 2015). After myocardial ischemia, oxidative stress causes cardiomyocytes to undergo apoptosis and necrotize, which triggers an inflammatory response in the heart. Inflammatory reactions are one of the characteristics of early reperfusion injury and an important cause of myocardial cell death (Zhou et al. 2021). MI/RI is a complex pathological process that can lead to the onset of sudden arrhythmia, an enlarged infarct area, and no reflow to the heart (Frank et al. 2012). The main mechanism driving oxidative stress and the cardiac inflammatory response is TLR4/NF-κB signaling. Interestingly, in the studies of geomagnetic activity and human health, some researchers have observed that geomagnetic activity is related to the inflammatory response of human blood vessels (Schiff et al. 2022). In exposure to a moderate strength (0.23–0.28 T) static magnetic field, the short-term (< 24 h) activation of IL-6 involved the coordinate upregulation of toll-like receptor-4 (TLR4) observed in human embryonic cells (Wang et al. 2009). TLR4 binds to its ligands and this, in turn, recruits the intracellular protein myeloid differentiation factor 88 (Myd88) and interacts with TNF receptor-associated factor 6 (TRAF-6) (Lee et al. 2016). These events mediate production of nuclear factor-κB (NF-κB), a large number of proinflammatory factors interleukin-1 (IL-1), interleukin-6 (IL-6), tumor necrosis factor-α (TNF-α), and monocyte chemotactic protein-1 (MCP-1) (Coutinho-Wolin Karen et al. 2022). All of these effects stimulate downstream inflammatory response such as oxygen free radical damage, increased lipid peroxide in plasma, decreased superoxide dismutase (SOD) and lactic dehydrogenase (LDH) expression in myocardial cells, and increased levels of malonaldehyde (MDA) (Hentia et al. 2018). Meanwhile, the expression of creatine kinase (CK) decreases and the function of myocardial cells is impaired (Rodriguez et al. 2003). As a result, the TLR4/NF-κB signaling pathway is crucial for controlling the inflammatory response during MI/RI. Currently, GMA is predictable similar to weather forecasting (Kim et al. 2014), and if we understand how it works, we can take more practical steps to treat the cardiovascular disease before geomagnetic storm. In order to better understand the effect of the geomagnetic activity on MI/RI, it is important to replicate the geomagnetic activity to identify its mechanism in MI/RI. Currently, Dst is frequently used as an indication of the relative strengths of geomagnetic activity. Geomagnetic activities are divided into five levels, i.e., quiet GMA (Dst <  − 20 nT), weak GMA (− 50 nT < Dst <  − 20 nT), moderate GMA (− 100 nT < Dst <  − 50 nT), major GMA (− 150 nT < Dst <  − 100 nT), and severe GMA (Dst <  − 150 nT) (Dimitrov et al. 2009).

In a previous work, we designed and established a simulated geomagnetic environment to transform GMA into a controllable experimental condition that is not affected by external factors (Wu et al. 2019). We performed an animal experiment and determined the effect of various GMA on MI/RI in rat models (Van Camp 2014). In this study, rat models of MI/RI were subjected to different levels of GMA stimulation to determine the effects of GMA on oxidative stress and inflammatory responses, as well as the potential links to the TLR4/NF-κB signaling pathway. According to the results of the research, we can analyze geomagnetic storm data in order to predict significant GMA in advance and to take proactive measures in preventing cardiovascular events.

## Materials and methods

### Chemical reagents

The research depended on 2,3,5-triphenyltetrazolium chloride (TTC, Sigma-Aldrich, USA). CK was detected using an automatic biochemical analyzer (Beckman AU480, USA). The LDH, SOD, and MDA were detected in rat serum using ELISA kits (Jiancheng, China). IL-1, IL-6, and TNF-α were detected in rat serum using ELISA kits (Huamei, China). TRIzol reagent (Invitrogen, USA) and cDNA Synthesis Kit (TaKaRa, Japan) and a MiniOpticon (Bio-Rad Laboratories, USA) were used for qPCR. All chemicals and solvents used were of either analytical or pharmaceutical grade.

### Equipment

The GMA platform used in this study has been reported in previous studies from our group and certified by a Chinese patent no. ZL201520208744.2 (Wu et al. 2019). Four separate parts make up this geomagnetic experimental platform, i.e., a metal shielding experiment cage, a radiation antenna, a programmable signal generator, and a control computer. Any combination of signal spectra, including analog Schumann resonances and geomagnetic bursts, can be produced by the signal generator under the control of the main control computer below 50 Hz. The metal shielding cage is 2 cm × 2 cm and 150 cm × 150 cm × 90 cm in size, and there is an opening on one side for the Helmholtz antenna. At the same time, the output signal can be set quickly on the computer. The calibration of the equipment uses LZT-6200 (Longzhentian, China) electromagnetic radiation detector. Dst is frequently used as an indication of the relative strengths of geomagnetic activity. Geomagnetic activities are divided into five levels as quiet GMA (Dst <  − 20 nT), weak GMA (− 50 nT < Dst <  − 20 nT), moderate GMA (− 100 nT < Dst <  − 50 nT), major GMA (− 150 nT < Dst <  − 100 nT), and severe GMA (Dst <  − 150 nT) (Dimitrov et al. 2009). In the experiments described in this work, the severe GMA group had a Dst index = 500 nt, the major GMA group had a Dst index = 150 nt, and the weak GMA group had a Dst index = 50 nt. The direction of the earth’s magnetic field is from south to north, and the direction of the magnetic field in the experiment is from north to south, so the Dst value is negative. A BIOPAC 150 electrophysiolograph was used where indicated.

### Animal experiments and ethics statement

Male-specific pathogen-free (SPF)-grade Sprague–Dawley (SD) rats weighing 200–220 g were obtained from the Laboratory Zoology Department of Kunming Medical University [license number SCXK (Yunnan) k2020-0006]. The animals were kept in a room-temperature environment (24 ± 1 °C) with a 12/12-h light/dark cycle. The rats were treated to adaptive feeding for a week while being given free access to rodent food and tap water. The treatments were carried out strictly in compliance with Chinese law governing the use and care of laboratory animals, and they were given the go-ahead by Kunming Medical University’s Medical Ethical Committee.

The MI/RI paradigm was developed as our group has previously explained (Wu et al. 2019). Using a heated surgical table, the rats’ body temperatures were kept at 37 °C following anesthesia with 2% isoflurane. A left thoracic incision was made to exteriorize the heart, and the left anterior descending coronary artery (LAD) was then wrapped in a slipknot of 6–0 silk suture and strung tightly. After 30 min of ischemia, the slipknot was untied, and the animal then underwent 24 h of reperfusion.

### Experimental groups

Rats were randomly divided into a control group which was housed in a general environment without intervention, three groups housed in severe, major, and weak GMA, or a GMA shielding environment. The rats in each group were further divided into sham operation groups (control) and operation groups (experimental) and were assigned to one of the following ten groups (*n* = 9 in each group) (see Table 1).

**Table 1:** Experimental groups

Geomagnetic activity place						Normal place		Shield place	
Severe GMA		Major GMA		Weak GMA		Sham	MI/RI	Sham	MI/RI
Sham	MI/RI	Sham	MI/RI	Sham	MI/RI				

### Myocardial infarct area measurements

Upon reperfusion, the heart’s tissue was divided into five equal parts below the ligation of the heart and parallel to the coronary groove. To assess the vitality of the cardiac tissue, all slices were weighed and incubated in 1% 2,3,5-triphenyltetrazolium chloride (TTC) for 15 min. The non-TTC stained area (white or pale, indicating infarct area) and TTC stained area (red, indicating non-infarct area) were examined using Image-Pro Plus image analysis software (Version 4.1, Media Cybernetics, LP, USA). The ratio of the infarcted myocardium to the whole myocardial tissue was calculated using the formula infarct size (%) = (infarct area/whole heart area) 100%.

### Left ventricular pressure test

Cardiac function was measured by left ventricular cannulation following anesthesia with 2% isoflurane. The left common carotid artery was isolated, followed by recording of the LVDP and ± dp/dtmax by electrophysiolograph (BIOPAC 150).

### Serum biochemistry

Blood collection was performed using an abdominal aortic artery catheter, followed by centrifugation at 3000 rpm for 15 min at 4 °C. To assess markers of cardiac muscle injury such CK and LDH, serum was isolated. An automatic blood biochemical analyzer (Beckman AU480, USA) was used to detect CK and LDH using system-specific reagents. Following the manufacturer’s instructions, an enzyme-linked immunosorbent assay (ELISA) was used to measure the levels of IL-1, IL-6, and TNF-α in serum. A microplate spectrophotometer was used to measure optical density (OD) at 450 nm, and cytokine concentration was determined by extrapolating to a standard curve.

### Hematoxylin and eosin (HE) staining

The rats were anesthetized 24 h after MI/RI and perfused with 4% paraformaldehyde. Next, the hearts were quickly taken out and left overnight to be fixed with 4% paraformaldehyde. The pieces were then deparaffinized in xylene and rehydrated using varying concentrations of alcohol. HE was then used to stain the pieces. With a light microscope, structural alterations in cardiac tissues were seen (Olympus, Tokyo, Japan).

### qRT-PCR

Total RNA was extracted from cardiac tissues using the TRIzol reagent. On the basis of cDNA sequences received from GenBank, oligonucleotide primer sequences were created (Table 2). Two microgram of total RNA was reverse transcribed into cDNA using a cDNA synthesis kit, and qPCR was carried out using the MiniOpticon qPCR detection equipment (Bio-Rad Laboratories). The relative quantification was calculated using the 2^−ΔΔCT^ method.

**Table 2:** Primers for qPCR analysis

Primers	Primer sequence (5′ to 3′)	Length of target sequence
TLR4-F	CCAGAGCCGTTGGTGTATCT	197 bp
TLR4-R	AGAAGATGTGCCTCCCCAGA	
MyD88-F	CGGAGGAGATGGGTTTCGAG	83 bp
MyD88-R	CCAGGCATCCAACAAACTGC	
TRAF6-F	CGCCAAAATGGAAACGCAGA	88 bp
TRAF6-R	TGCTTCCATCTCGGCAACTT	
NF-κB-F	CATACGCTGACCCTAGCCTG	135 bp
NF-κB-R	TTTCTTCAATCCGGTGGCGA	
TNF-α-F	TTCTCATTCCTGCTCGTGGC	112 bp
TNF-α-R	AACTGATGAGAGGGAGCCCA	
MCP-1-F	TGATCCCAATGAGTCGGCTG	127 bp
MCP-1-R	TGGACCCATTCCTTATTGGGG	
*β*-actin-F	GCTGTGCTATGTTGCCCTAGAC	118 bp
*β*-actin-R	CCGCTCATTGCCGATAGTGATG	

### Western blotting

Using a BCA measurement kit, tissue protein extracts were prepared and measured. SDS-PAGE was used to separate a total of 30 μg of protein, which was then electro-transferred onto PVDF membrane (Millipore, USA). The membrane was incubated overnight at 4 °C with primary antibodies such as anti-TLR4 (1:1000, ab13867, Abcam), anti-MyD88 (1:1000, 4283, Cell Signaling Technology), anti-TRAF6 (1:800, ab33915, Abcam), anti-NF-κB (1:1000, 8242, Cell Signaling Technology), anti-TNF-α (1:1000, 11,948, Cell Signaling Technology), anti-MCP1 (1:1000, ab25124, Abcam). The membranes were then exposed to HRP-conjugated goat anti-rabbit IgG (1:2000, ab6721, Abcam) for 2 h at room temperature. Band intensity was normalized to GAPDH and analyzed using Image Lab v5.2 software.

### Statistical analysis

The data are expressed as the means ± SEM. Analysis and plotting were performed using SPSS (version 20; IBM-SPSS Inc, Armonk, NY) and GraphPad Prism9 (San Diego, US). A comparison between the two groups was statistically detected using Student’s *t*-test, and more than two groups were evaluated by one-way ANOVA followed by Tukey’s post hoc test. When *p* < 0.05, there were significant differences between the data.

## Results

### Effects of simulated geomagnetic activity on myocardial infarction area in the MI/RI rats

**Fig. 1:**
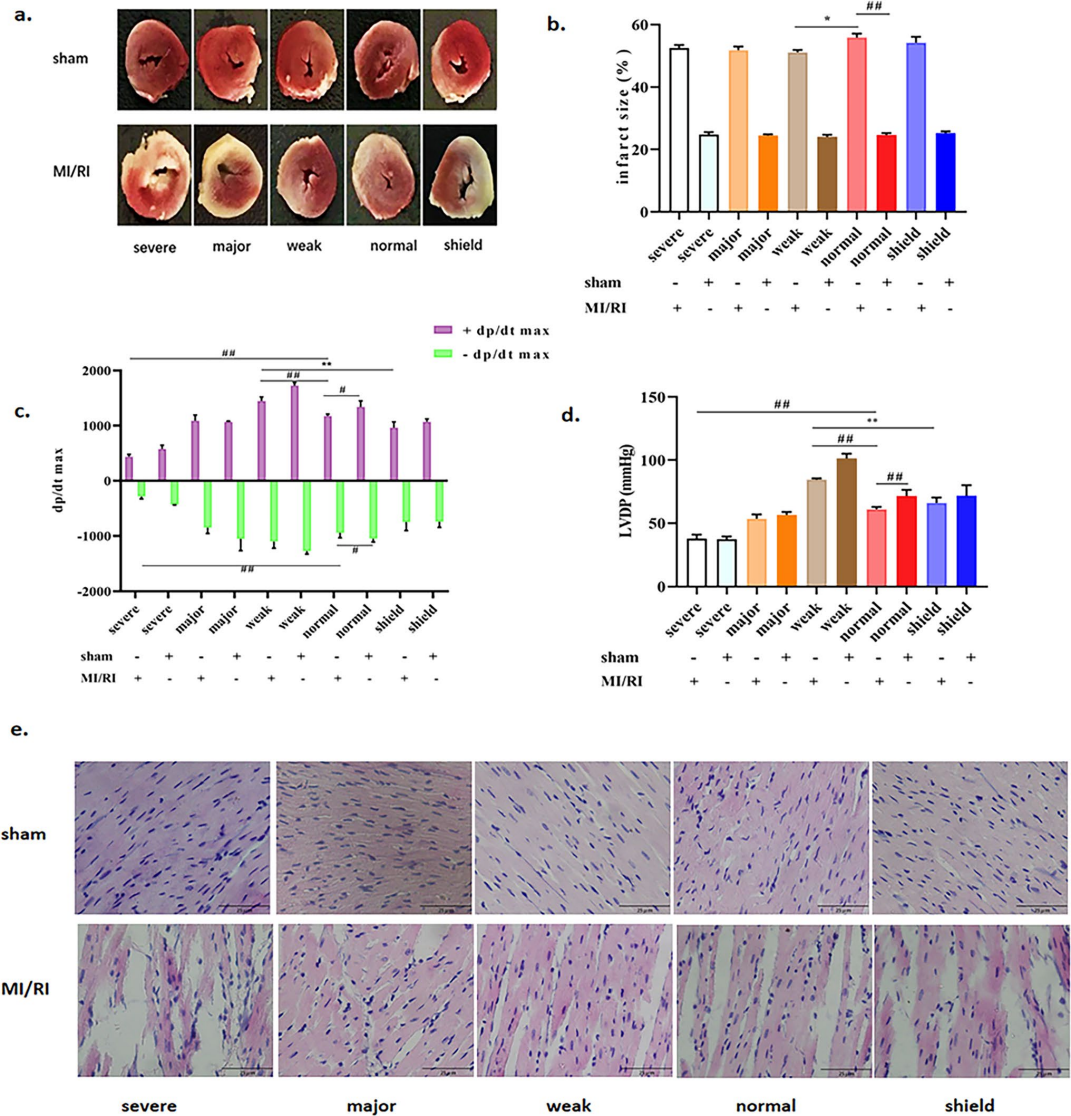
**a** Representative heart sections stained with TTC. **b** Quantitative analysis of the infarct size in each group. **c** Statistics of cardiac ± dp/dt max. **d** Statistics of LVDP measurements. **e** Pathological changes in the heart tissue was detected using HE staining (160 × , scale bar = 25 μm). Data were expressed as the means ± SEM (*n* = 6). ^#^*p* < 0.05, ^##^*p* < 0.01 vs. the MI/RI group; ^**^p < 0.01 vs. the weak GMA group

The success of the rat MI/RI model was confirmed by the TTC staining results, which showed that the infarct area in the MI/RI group was substantially increased as compared to rats in the sham group (*p* < 0.01). In contrast, rats suffering from MI/RI injury had their infarct size restricted by modest GMA simulation exposure (*p* < 0.05) (Fig. 1a, b).

### Effects of simulated geomagnetic activity on ± dp/dtmax and LVDP in the MI/RI rat model

To verify the effects of different intensities of GMA on cardiac function in the MI/RI rat model, we examined ± dp/dtmax and LVDP in these rats. Compared with the control MI/RI group, the + dp/dtmax and LVDP of rats in the weak GMA group were elevated, and the ± dp/dtmax and LVDP of rats in the severe GMA group were reduced (*p* < 0.01). Meanwhile, compared with the weak GMA group, the ± dp/dtmax and LVDP of the shield + MI/RI group were reduced (*p* < 0.01) (Fig. 1c, d). These results indicated that weak GMA can improve the cardiac function in the MI/RI rat model, while severe GMA can exacerbate cardiac injury.

### The effects of differing geomagnetic activity intensity on myocardial fiber morphology

We conducted HE staining of sectioned cardiac tissue to observe myocardial fiber morphology. As shown in Fig. 1e, in the sham operation group, we found that the myocardial fibers were closely connected and there were few heterotypic cells observed. Moreover, these cells showed that the nucleus was spindle shaped and the boundary of cell membranes was clear. In the MI/RI group, the myocardial fibers were broken, the number of heteromorphic cells was increased, and the cell membrane boundary was blurred. Compared with the MI/RI group, the myocardium damage area of rats and the atypia cells in the weak GMA + MI/RI group was decreased, while the myocardium damage area of rats and the atypia cells in the severe GMA + MI/RI group were increased. The HE staining showed that the weak GMA could limit damage to myocardial cells, while the severe GMA promoted more adverse effects.

### Effects of simulated geomagnetic activity on myocardial enzyme markers in the MI/RI rat model

**Fig. 2:**
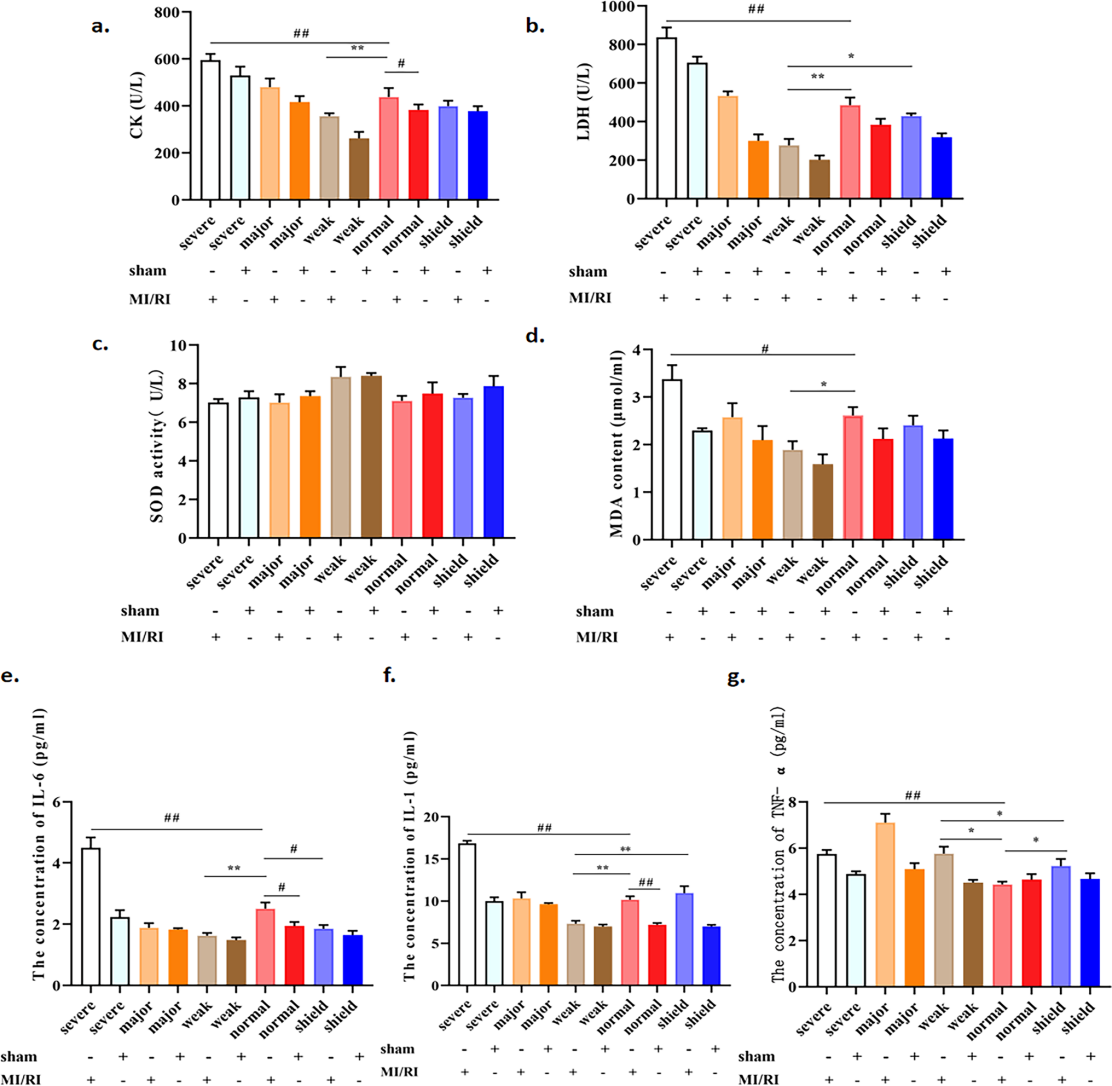
Effects of simulated geomagnetic activity on myocardial enzymes, oxidative stress markers, and inflammatory cytokines in the MI/RI rat model. **a** Effects of GMA on CK activity in MI/RI rat models. **b**–**d** Effect of GMA on LDH, SOD, and MDA levels in the MI/RI rat model. **e**–**g** Effect of GMA on IL-1, IL-6, and TNF-α activity in MI/RI rats. Data were expressed as the means ± SEM (*n* = 6). ^#^*p* < 0.05, ^##^*p* < 0.01 vs. the MI/RI + normal group; ^*^p < 0.05, ^**^p < 0.01 vs. the weak GMA group

According to Fig. 2a, b, the two main clinical indicators of myocardial damage were CK and LDH. When compared to the sham group, the MI/RI group had significantly higher serum levels of CK (*p* < 0.01). A weak GMA dramatically reduced the MI/RI-induced levels of both CK and LDH (*p* < 0.01). However, severe GMA increased levels of CK and LDH in the MI/RI rat model (*p* < 0.01). Meanwhile, compared with the weak GMA group, the levels of LDH in the shield + MI/RI group were increased (*p* < 0.05). These results indicated that weak GMA can protect the MI/RI rat model from clinical signs of MI/RI and that this effect can be limited by GMA shielding. Moreover, severe GMA further potentiated harmful effects stemming from MI/RI.

### Effects of simulated geomagnetic activity on serum oxidative stress markers in the MI/RI rat model

We measured the levels of SOD and MDA in the serum of MI/RI rats. As shown in Fig. 2c, d, the activity of SOD the MI/RI group was lower than that measured in the sham group. Compared with the MI/RI group, the level of SOD in the weak GMA group was higher. The level of MDA in the MI/RI group was increased compared with that in the sham group. Weak GMA exposure significantly decreased MDA levels, and severe GMA increased the level of MDA (*p* < 0.05). Taken together, these results suggest that weak GMA can suppress oxidative stress induced by MI/RI, whereas the results of severe GMA had the opposite effect.

### Effects of simulated geomagnetic activity on serum inflammatory cytokines in the MI/RI rat model

Inflammatory cytokines were a key indicator of MI/RI injury. As shown in Fig. 2e–g, there were measurable increases in serum levels of IL-1, IL-6, and TNF-α in the MI/RI group. Weak GMA exposure dramatically reduced the MI/RI-induced levels of both IL-1 and IL-6 (*p* < 0.01); however, severe GMA increased serum levels of IL-1 and IL-6 in the MI/RI rat model (*p* < 0.01). Compared with the normal environment, the GMA shielding environment reduced IL-6 levels (*p* < 0.05) and increased TNF-α levels (*p* < 0.05). Compared with the MI/RI group, the level of TNF-α in the weak GMA group was increased (*p* < 0.01). Of note, when compared with the weak GMA group, the level of IL-1 in the shield + MI/RI group was increased, and TNF-α was decreased (*p* < 0.05). These results suggest that weak GMA can reduce inflammatory markers following MI/RI, whereas the results of severe GMA had the opposite effect.

### Effects of simulated geomagnetic activity on TLR4/NF-κB signaling in the MI/RI rat model

**Fig. 3:**
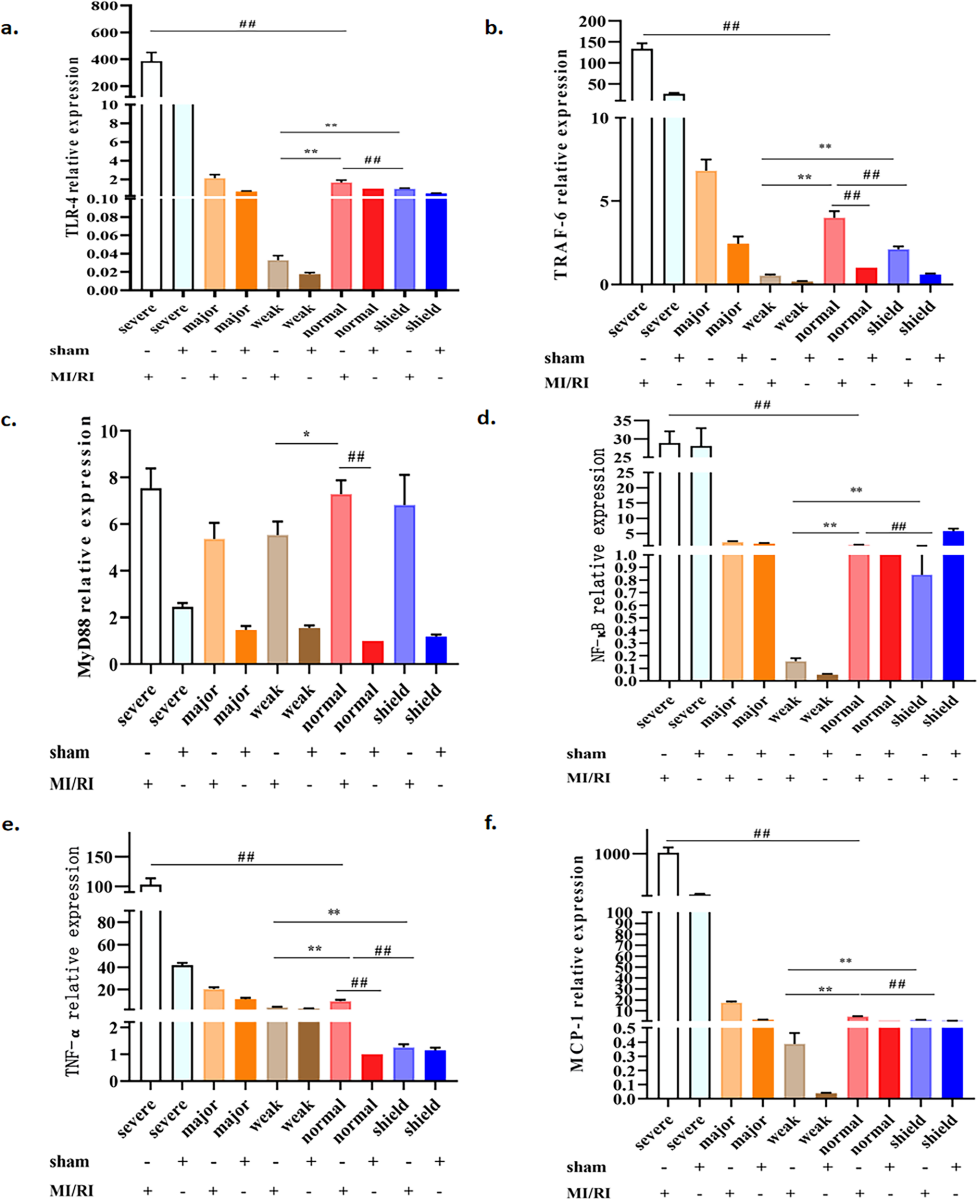
Effects of simulated GMA on mRNA levels of TLR4/NF-κB signaling pathway genes in the MI/RI rat model. **a**–**f** Statistics of TLR-4, TRAF-6, MyD88, NF-κB, TNF-α, and MCP-1 mRNA expression. The data were expressed as the means ± SEM (*n* = 6). ^#^*p* < 0.05, ^##^*p* < 0.01 vs. the MI/RI group; ^**^p < 0.01 vs. the weak GMA group

The potential regulatory mechanism underlying the protective function of the simulated GMA in the MI/RI injury model was the next area of research. We considered that TLR4/NF-κB-mediated inflammatory signaling was associated with MI/RI injury and this led us to analyze mRNA expression of key markers associated with activation of this pathway. qPCR analysis (Fig. 3) indicated that *TLR4*, *TRAF-6*, *MyD88*, *NF-κB*, *TNF-α*, and *MCP-1* transcripts were clearly decreased in the weak GMA group, compared with the MI/RI group (*p* < 0.05, *p* < 0.01). Notably, the decreased expression of *TLR4*, *TRAF-6*, *NF-κB*, *TNF-α*, and *MCP-1* induced by MI/RI was significantly reversed by exposure to severe GMA (*p* < 0.05, *p* < 0.01). Compared with the weak GMA group, the expression *of TLR4*, *TRAF-6*, *NF-κB*, *TNF-α*, and *MCP-1* in the shield + MI/RI group was increased (*p* < 0.05, *p* < 0.01). Compared with the normal group, the expressions of *TLR4*, *TRAF-6*, *NF-κB*, *TNF-α*, and *MCP-1* in the shield + MI/RI group were also decreased (*p* < 0.05, *p* < 0.01). These results indicated that weak GMA and GMA shielding environment significantly alleviate expression of MI/RI-induced inflammatory markers that might be upregulated through the TLR4/NF-κB signaling axis, but severe GMA increased expression of MI/RI-induced inflammatory markers that might be upregulated through the TLR4/NF-κB signaling axis.

### Effects of simulated geomagnetic activity on the level of key protein markers of the TLR4/NF-κB signaling pathway in the MI/RI rat model

**Fig. 4:**
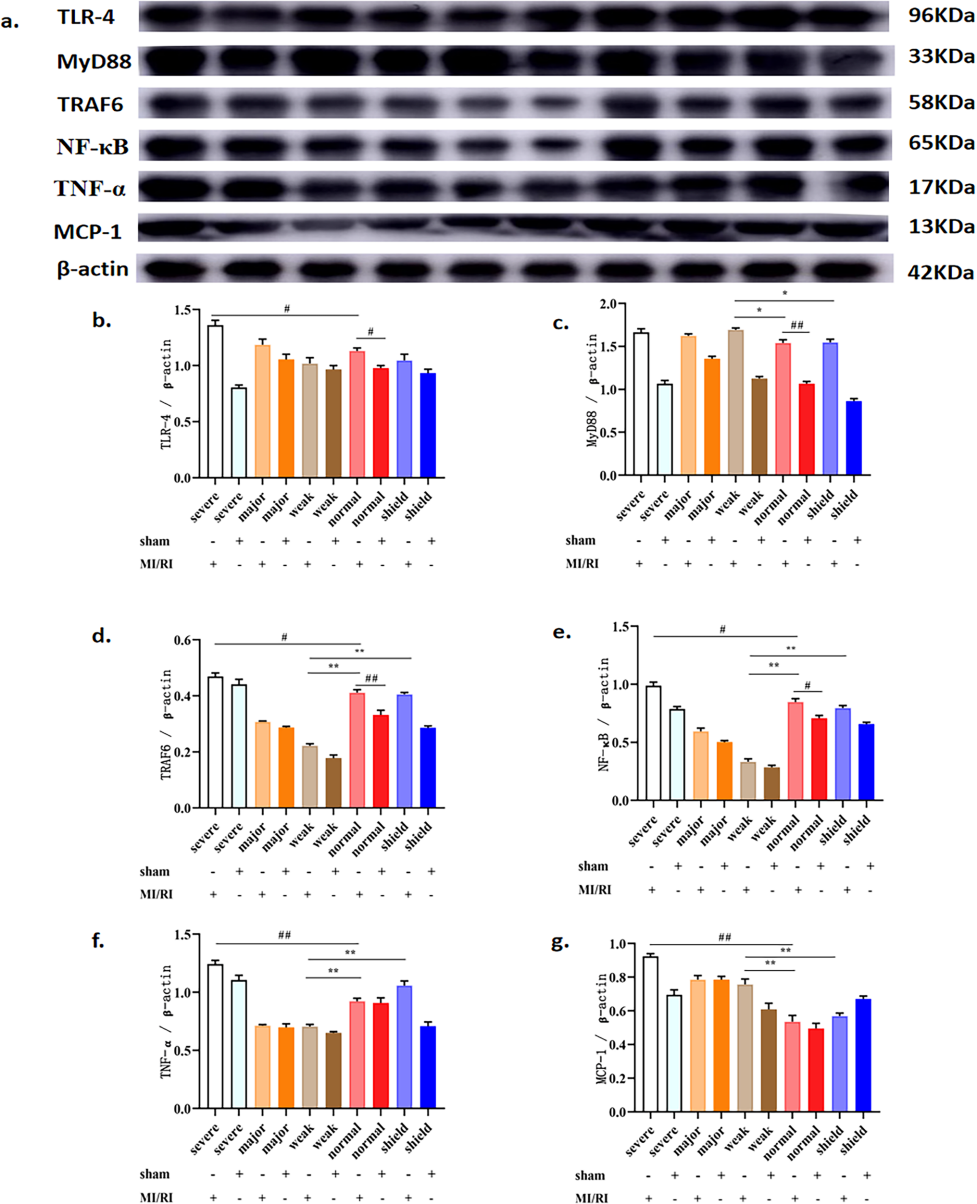
Effects of simulated GMA on protein level of the TLR4/NF-κB signaling pathway in the MI/RI rat model. **a** Protein band map of TLR-4, TRAF-6, MyD88, NF-κB, TNF-α, and MCP-1. **b**–**g** Statistics of TLR-4, TRAF-6, MyD88, NF-κB, TNF-α, and MCP-1 protein abundance. The data are expressed as the means ± SEM (*n* = 6). ^#^*p* < 0.05, ^##^*p* < 0.01 vs. the MI/RI group; ^*^p < 0.05, ^**^p < 0.01 vs. the weak GMA group

To evaluate the potential relationship between the effects of simulated GMA and limited activation of the TLR4/NF-κB signaling axis, the protein levels of TLR4, TRAF-6, MyD88, NF-κB, TNF-α, and MCP-1were evaluated by western blotting. As shown in Fig. 4, compared with that in the MI/RI group, the expression of MyD88 and MCP-1 was increased in the weak GMA group. In contrast, the expression of TRAF-6, NF-κB, and TNF-α in the weak GMA group was decreased (*p* < 0.05). Meanwhile, the expression of TLR4, TRAF-6, NF-κB, TNF-α, and MCP-1 induced by MI/RI was significantly increased by severe GMA (*p* < 0.05). Compared with the weak GMA group, the expression of MyD88 and MCP-1 was decreased, and TRAF-6, NF-κB, TNF-α, and MCP-1 were increased in the shield + MI/RI group (*p* < 0.05). Compared with the normal group, there was no significant difference in the expression of TLR4, TRAF-6, NF-κB, TNF-α, and MCP-1 in the shield + MI/RI group. These results indicated that weak GMA might significantly alleviate MI/RI-induced inflammation through an attenuation of TLR4/NF-κB signaling, whereas severe GMA might significantly aggravate MI/RI-induced inflammation through an amplification of TLR4/NF-κB signaling.

## Discussion

The study showed that GMA was linked to major cardiac enzymes (CK and LDH), oxidation levels, inflammatory markers, and the TLR4/NF-κB signaling pathway. CK was one of the markers of myocardial injury, and increases in serum LDH were utilized as an auxiliary diagnostic sign in the later stages of an acute myocardial infarction (Horjus et al. 2011; Ndrepepa and Kastrati 2018; Zhang et al. 2022). Results obtained in this study showed that the levels of CK and LDH were significantly increased after MI/RI, while the levels of CK and LDH were decreased after weak GMA intervention. In contrast, severe GMA intervention was found to aggravate myocardial damage. Sert et al. (2010) reported an enhancement in the values of CK-MB after exposure to the magnetic field (1.5 T), which was similar with our findings. Although the severe GMA was 500 nT, it was substantially lower than 1.5 T. Overall, results gathered showed that weak GMA had a protective effect on MI/RI-induced injury in rats and severe GMA aggravated MI/RI-induced injury in rats.

A significant amount of oxygen free radicals was produced during periods of myocardial anoxia; increased SOD expression was used as an indicator of oxidative stress response (Gao et al. 2022; Huang et al. 2015). MDA levels were also an important marker of oxidative stress response damage (Sun et al. 2021). Although enormous evidences showed that magnetic field affected ROS levels, there was no consensus about their exact effects (Ghodbane et al. 2013). It was well acknowledged that ROS level was dependent on the dynamic balance between ROS generation and elimination, but only a few studies explored whether the production or elimination process of ROS was affected by magnetic field (Sies 2017, Sies et al. 2017). In this study, SOD activity was decreased and MDA content increased after MI/RI, indicative of an oxidative stress response. The SOD abundance in the experimental group with weak GMA intervention was significantly increased, and the MDA content was reduced, indicating that weak GMA could alleviate the oxidative stress within myocardial cells as a result of MI/RI and thus protect myocardial cells from damage. On the contrary, severe GMA enhanced oxidative stress, leading to a decrease of SOD, an increase in MDA, and the aggravation of myocardial cell damage. The SOD activity in the rabbits’ serum was significantly increased in electromagnetic radiation with a frequency of 900 MHz from a cellular telephone (Kemal Irmak et al. 2002); the radiation of cellular telephone was higher than severe GMA. Magnetic field exposure caused mild oxidative stress (modest ROS increases and changes in antioxidant levels) and possibly activated anti-inflammatory processes (decrease in proinflammatory and increase in anti-inflammatory cytokines).

Meanwhile, MI/RI was accompanied by inflammatory response; inhibiting inflammatory response had become important in reducing the prognosis of MI/RI (Wang and Lin 2020). Our results showed that weak GMA significantly reduced the levels of myocardial inflammatory factors IL-1, IL-6, and TNF-α in the MI/RI rat model, and severe GMA significantly increased the levels of myocardial inflammatory factors IL-1, IL-6, and TNF-α in the MI/RI rat model. Similarly, Wang et al. (2009) demonstrated that exposure to moderate strength (0.23–0.28 T) static magnetic field, IL-6 involved the coordinate upregulation of TLR4 was observed in human embryonic cells (Frank et al. 2012). These findings suggested that weak GMA attenuated MI/RI-mediated myocardial inflammatory response, whereas severe GMA exacerbated it.

The activation of the TLR4/NF-κB signaling pathway regulated the release of downstream inflammatory factors and thus mediates the inflammatory response (Gunther et al. 2018). In this research, the mRNA and protein expression of key targets in the pathway was examined, which indirectly verified the effect of GMA on MI/RI through the regulation of the TLR4/NF-κB signaling pathway. As a result of weak GMA intervention, the expression of TLR4, TRAF6, NF-κB, and TNF-α was significantly decreased, the expression of MyD88 and MCP-1 was increased, the inflammatory response was blunted, the area of myocardial infarction was reduced, and cardiac function was improved. But after the intervention of severe GMA, the opposite result was observed, and similar to the short-term (< 24 h) activation of IL-6 involved the coordinate upregulation of TLR4 was observed in human embryonic cells (Frank et al. 2012). In GMA shielding environment, the mRNA expression of TLR4, TRAF-6, NF-κB, TNF-α, and MCP-1 was decreased, and the IL-6 in serum also decreased, but there was no difference in the protein expression compared with the normal group. These results indirectly indicate that the TLR4/NF-κB signaling pathway might be involved in a MI/RI protective effect stemming from GMA intervention. Weak GMA limited the inflammatory response caused by MI/RI and reduced the damage of myocardial tissue, while severe GMA further promoted this damage. The GMA shielding environment had limitations on the inflammatory response induced by MI/RI, in terms of mRNA expression.

## Conclusions

The study’s findings revealed a bidirectional regulatory effect of geomagnetic activity on the inflammatory response of MI/RI rats, that is, weak GMA had a limiting effect on the inflammatory response caused by MI/RI, thereby protecting the injury of MI/RI, and severe GMA had a stimulating effect on the inflammatory response caused by MI/RI, thereby aggravating the injury of MI/RI. Thus, it opens up a new way for clinical prevention and treatment of these conditions. The increased CVD mortality and morbidity during geomagnetic storms might be reasonably explained based on the effect of severe GMA on the inflammatory response. From a protective perspective, the establishment of a weak GMA environment, such as within hospital settings or bedrooms, holds promise in safeguarding individuals with cardiovascular ailments. On the other hand, considering the potential exacerbation of damage, the proactive monitoring of geomagnetic storm data enables the prediction and prevention of adverse GMA effects on cardiovascular patients. Notably, it was critical to acknowledge the potential limitation of this study that TLR4 agonists and inhibitors were not used during the experiment, so the experimental results might demonstrate the effect of GMA on TLR4 signaling. In particular, it focused only on a 24-h period while overlooking other timeframes. Findings obtained from a specific time point fall short of fully elucidating the comprehensive impact of geomagnetic activity on MI/RI, necessitating a comprehensive examination of the temporal component.

## Data Availability

The datasets generated and/or analyzed during the current study are available from the corresponding author on reasonable request in compliance with ethical standards.
